# MnCaTa_2_O_7_—A Magnetically Ordered Polar Phase Prepared via Cation Exchange

**DOI:** 10.1021/acs.chemmater.3c01850

**Published:** 2023-09-09

**Authors:** Subhadip Mallick, Fabio Orlandi, Pascal Manuel, Weiguo Zhang, P. Shiv Halasyamani, Michael A. Hayward

**Affiliations:** † Department of Chemistry, Inorganic Chemistry Laboratory, University of Oxford, South Parks Road, Oxford OX1 3QR, UK; ‡ ISIS Facility, Rutherford Appleton Laboratory, Chilton, Oxon OX11 0QX, UK; § Department of Chemistry, University of Houston, 112 Fleming Building, Houston, Texas 77204-5003, United States

## Abstract

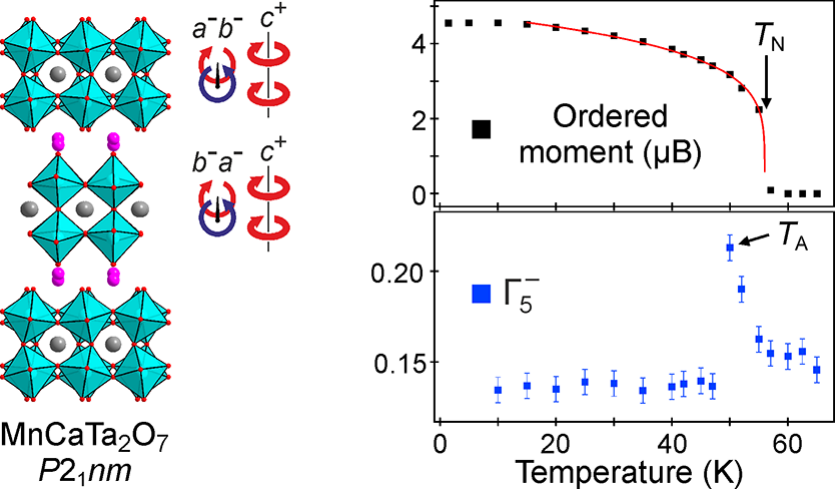

Reaction between the pseudo-Ruddlesden-Popper phase Li_2_CaTa_2_O_7_ and MnCl_2_ at 375 °C yields MnCaTa_2_O_7_, a paramagnetic polar phase (space group *P*2_1_*nm*), which adopts an *a*^–^*b*^–^*c*^+^/*b*^–^*a*^–^*c*^+^ distorted, layered perovskite structure. Magnetization and neutron diffraction data show that MnCaTa_2_O_7_ adopts an antiferromagnetically ordered state below *T*_N_ = 56 K and exhibits large lattice parameter anomalies and a transient increase in its polar distortion mode at *T*_A_ = 50 K. However, in contrast to the related phase MnSrTa_2_O_7_, MnCaTa_2_O_7_ shows no strong signature of weak ferromagnetism and thus shows no signs of magnetoelectric coupling. The differences in physical behavior between the two Mn*A*Ta_2_O_7_ phases appear to be related to their differing Mn cation-order and differing TaO_6_ tilting schemes and demonstrate that even subtle changes to these orderings can have large effects on the distortion-mode couplings, which drive complex behavior of this class of “hybrid improper” ferroelectric material.

## Introduction

Magnetoelectric multiferroic materials simultaneously exhibit a spontaneous, switchable electrical polarization (ferroelectricity) and a spontaneous, switchable magnetic polarization (ferromagnetism)^1,2^ and are highly desired because they could allow the construction of novel devices with applications in digital data manipulation and storage.^3,4^ However, the preparation of novel materials which display magnetoelectric behavior, is challenging. This is principally because materials can only exhibit ferroelectric behavior if they adopt noncentrosymmetric (NCS) crystal structures,^5,6^ and unfortunately, asymmetric packing schemes tend to be thermodynamically disfavored compared to centrosymmetric alternatives, making ferroelectric behavior, and thus magnetoelectric behavior, rare.

Conventionally, ferroelectric behavior has been introduced into materials, by utilizing electronically driven structural distortions which break the inversion symmetry of a phase. Typically, these symmetry-breaking distortions are driven by a second-order Jahn-Teller (SOJT) instability^7^ arising from the inclusion of either octahedrally coordinated d^0^ transition-metal cations (e.g., Ti^4+^ in BaTiO_3_)^8,9^ or *n*s^2^ posttransition-metal cations (e.g., Pb^2+^ in PbZrO_3_),^10,11^ which, in favorable circumstances, yield NCS crystal structures and induce polar behavior.

While the use of SOJT distortions has been successful in preparing a range of polar phases, the need to include d^0^ and/or *n*s^2^ cations limits the chemical diversity of polar materials and the “closed shell”, diamagnetic electronic configurations of these ions disfavors magnetism.^12^ Recently, an alternative “trilinear-coupled hybrid-improper” mechanism for stabilizing NCS structures has been receiving attention.^13^ In this stabilization scheme, two nonpolar distortions (typically the low-energy octahedral tilting distortions of layered perovskites phases) couple together to stabilize a third polar distortion mode, which is unstable in the absence of the stabilizing nonpolar distortions. As the polar distortion mode is not the primary order parameter of the ferroelectric phase transition, materials of this type are often referred to as “hybrid improper” ferroelectrics.^13−15^

As noted above, in contrast to the SOJT stabilization of NCS phases, the trilinear-coupled hybrid-improper mechanism does not rely on the electronic features of a phase (i.e., d^0^ or *n*s^2^ ion configurations). Thus, in principle, it places no restrictions on the chemical composition of polar materials of this type, facilitating the inclusion of paramagnetic ions in polar materials. Indeed, one of the first materials predicted to exhibit trilinear-coupled ferroelectric behavior, Ca_3_Mn_2_O_7_, adopts a weak ferromagnetic state at low temperature, which is observed to be magnetoelectrically coupled to the electrical polarization of the phase.^13^

However, further investigation of the layered Ruddlesden-Popper^16−21^ and Dion-Jacobson^22−25^ oxide phases, which exhibit hybrid improper ferroelectric behavior, reveals that this class of materials does not have as much “chemical freedom” as it may appear at first sight.^15^ This is because the hybrid improper mechanism utilizes a coupling between two distinct octahedral tilting distortions. This requires phases with highly distorted structures that are only stable in layered perovskite materials with small Goldschmitt tolerance factors, estimated to be *t* <0.87.^15,17^ Given that the tolerance factor, *t*, is defined as *t* = <*A*–O> / (√2 × <*B*–O>),^26^ sufficiently distorted frameworks are only stable in compounds with small *A*-cations and large *B*-cations. This condition acts to exclude a large number of *A*/*B* cation combinations from exhibiting hybrid improper ferroelectric behavior, including the majority of preparable Ruddlesden-Popper and Dion-Jacobson oxides with paramagnetic cations on the B-site.^15^

To address this limitation, we have been using the facile cation-exchange chemistry of *A*′*AB*_2_O_7_ Dion-Jacobson^27−29^ and Li_2_*AB*_2_O_7_ pseudo-Ruddlesden-Popper oxides^30^ to prepare metastable layered perovskite phases with small tolerance factors, to investigate the possibility of trilinear-coupled hybrid-improper ferroelectric behavior in these materials. Building on this work, we have demonstrated that paramagnetic Mn^2+^ cations can be exchanged for Li^+^ ions in the pseudo-Ruddlesden-Popper oxide Li_2_SrTa_2_O_7_, to yield MnSrTa_2_O_7_, a paramagnetic polar phase that exhibits signatures of magnetoelectric coupling at low temperature.^31^ Here, we describe a study of the related phase, MnCaTa_2_O_7_, prepared via an analogous cation-exchange reaction from Li_2_CaTa_2_O_7_.

## Experimental Section

### Synthesis of Li_2_CaTa_2_O_7_

Polycrystalline samples of Li_2_CaTa_2_O_7_ were prepared by a ceramic synthesis method from Ta_2_O_5_ (99.9985%, dried at 900 °C), CaCO_3_ (99.8%), and Li_2_CO_3_ (99.998%). Suitable stoichiometric ratios of Ta_2_O_5_ and CaCO_3_ were ground together in an agate pestle and mortar and combined with a 3% excess of Li_2_CO_3_ (to compensate for loss due to volatilization at high temperature). These mixtures were placed in an alumina crucible and heated at 800 °C in the air for 12 h. The mixtures were then reground, pressed into 13 mm pellets, and heated at 1200 °C for two periods of 2 h. The samples were reground between heating cycles, pressed into pellets, placed in an alumina crucible, and directly put in a furnace, which was kept at 1200 °C.

### Preparation of MnCaTa_2_O_7_

MnCaTa_2_O_7_ was prepared via a cation exchange reaction from Li_2_CaTa_2_O_7_. Li_2_CaTa_2_O_7_ was ground together with anhydrous MnCl_2_ in a 1:5 molar ratio using an agate pestle and mortar in an argon-filled glove box. The mixture was then heated at 375 °C for 3 days under flowing argon. The reaction mixture was then washed with distilled water to remove the remaining chlorides.

Magnetization data, described below, showed that samples of MnCaTa_2_O_7_ contained small quantities of Mn_3_O_4_ (a ferrimagnet, *T*_N_ ∼43 K^32,33^) not detectable by diffraction. To remove this magnetic impurity, a sample was heated to 350 °C under a flow of 10% H_2_ in argon, to reduce the Mn_3_O_4_ to MnO. SXRD data collected after this reduction process showed no changes with respect to the initial material.

### Characterization

X-Ray powder diffraction data were collected using a PANalytical X’pert diffractometer incorporating an X’celerator position-sensitive detector (monochromatic Cu Kα_1_ radiation). High-resolution synchrotron X-ray powder diffraction (SXRD) data were collected using the I11 instrument at the Diamond Light Source Ltd. Diffraction patterns were collected using Si-calibrated X-rays with an approximate wavelength of 0.825 Å from samples, sealed in 0.3 mm diameter borosilicate glass capillaries. Time-of-flight neutron powder diffraction (NPD) data were collected using the GEM diffractometer (ambient temperature) and WISH diffractometer^34^ (low temperature) located at the ISIS neutron source, from the samples loaded in vanadium cans. Rietveld refinements were performed using the TOPAS Academic (V6) package.^35^ Second harmonic generation (SHG) response of samples was measured from powder samples with the SHG intensity compared to a standard sample of α-SiO_2_. No index matching fluid was used in any of the experiments. A detailed description of the experimental setup and process has been reported previously.^36^ DC magnetization data were collected using a Quantum Design MPMS SQUID magnetometer from samples contained in gelatin capsules.

## Results

### Structural Characterization of Li_2_CaTa_2_O_7_

SXRD data collected from Li_2_CaTa_2_O_7_ could be indexed by an orthorhombic unit cell (*a* = 5.51225(3) Å, *b* = 5.46373(3) Å, *c* = 18.2321(1) Å) with reflection conditions consistent with the *Pna*2_1_ (#33) space group previously reported for the phase.^37,38^ Thus, a model based on the previously reported structure of Li_2_CaTa_2_O_7_ was refined against the SXRD data to achieve a good fit (wRp = 1.32%; *R*_Bragg_ = 1.43%) yielding a structural model in good agreement with previous reports,^37^ as described in detail in the Supporting Information.

### Structural Characterization of MnCaTa_2_O_7_

SXRD and NPD data (GEM) collected from the manganese exchanged sample of Li_2_CaTa_2_O_7_, henceforth referred to as MnCaTa_2_O_7_, could be indexed using a primitive orthorhombic unit cell (*a* = 5.5155(4) Å, *b* = 5.5169(4) Å, *c* = 19.0303(12) Å) consistent with a √2 × √2 × 1 geometric expansion of an aristotype *n* = 2 Ruddlesden-Popper framework. Powder SHG measurements reveal that MnCaTa_2_O_7_ is SHG active (0.45 times α-SiO_2_), indicating the phase adopts a noncentrosymmetric crystal structure. A symmetry analysis of the structural tilting distortions of *n* = 2 Ruddlesden-Popper phases^27,39^ revealed two candidate noncentrosymmetric structures with space group symmetries consistent with the reflection conditions observed in the diffraction data: *P*2_1_*nm* (#31) and *P*2*cm* (#28), which exhibit *a*^–^*b*^–^*c*^+^/*b*^–^*a*^–^*c*^+^ and *a*^–^*b*^–^*c*^+^/–(*a*^–^*b*^–^)*c*^+^ tilting distortions, respectively. Structural models were constructed to describe these two distorted frameworks and refined against the diffraction data.

There are two crystallographically distinct interlayer tetrahedral coordination sites in both the *P*2_1_*nm* and *P*2*cm* symmetry models, allowing both Mn site-ordered and site-disordered structures to be described by both the *P*2_1_*nm* and *P*2*cm* models. Initially, in refinements against PND data, the manganese cations were distributed across both sites to describe site-disordered Mn cation arrangements in both models. However, refinement of the Mn site occupancies led to the majority of the Mn cations (>98%) being located on one of the sites in the *P*2_1_*nm* symmetry model, accompanied by a large improvement in the fit to the data. Thus, a Mn cation-ordered model was adopted for the *P*2_1_*nm* symmetry model. In contrast, the *P*2*cm* symmetry model retained a Mn site-disordered arrangement when the Mn site occupancies were refined. The difference in refined Mn site-order between the *P*2_1_*nm* and *P*2*cm* symmetry models can be rationalized by observing that the two crystallographically distinct Mn sites in the *P*2_1_*nm* symmetry model are arranged as a chequerboard, while the analogous sites in the *P*2*cm* symmetry model are arranged in stripes.

**Figure 1:**
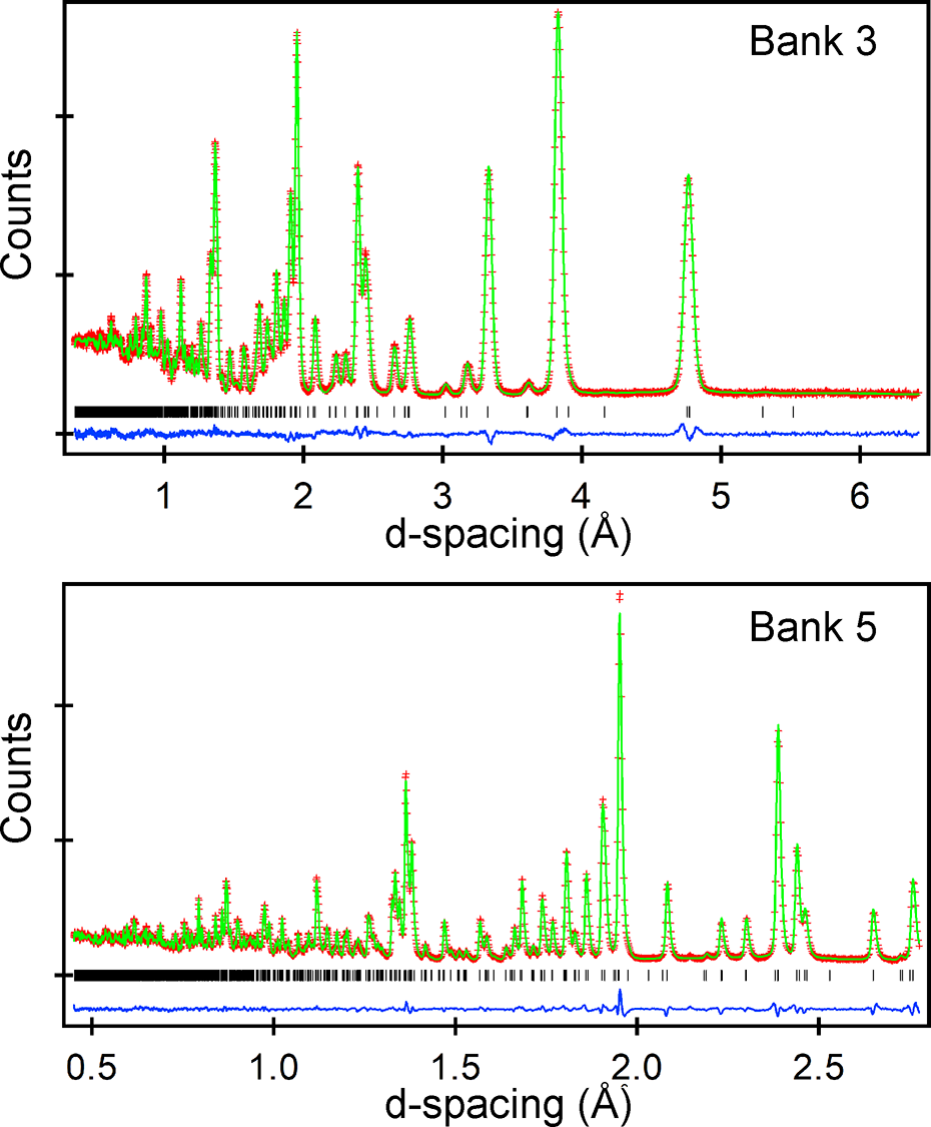
Observed, calculated, and difference plots from the structural refinement of MnCaTa_2_O_7_ against NPD data (GEM) collected at room temperature. Data from detector banks 3 (2θ = 35°) and 5 (2θ = 91°) are shown. Data from all detector banks are shown in the Supporting Information.

**Figure 2:**
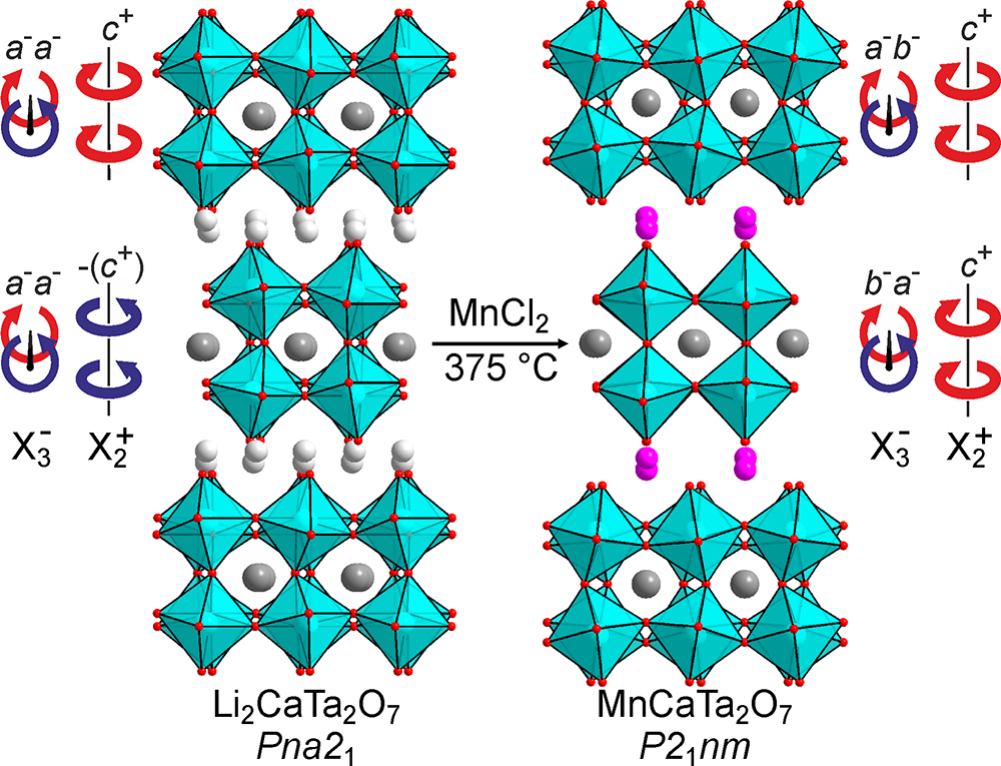
Crystal structures of Li_2_CaTa_2_O_7_ and MnCaTa_2_O_7_. Light gray, dark gray, purple, blue and red spheres represent Li, Ca, Mn, Ta, and O, respectively. Arrows indicate octahedral tilting schemes.

On convergence, it was clear that the *P*2_1_*nm* symmetry model gave a much better fit to the NPD data (wRp = 2.87%, Rp = 3.08%) compared to the *P*2*cm* symmetry model (wRp = 8.58%, Rp = 5.75%), indicating that the structure of MnCaTa_2_O_7_ is best described by the *P*2_1_*nm* symmetry model. On convergence, the Mn site occupancy refined to 1.01(1) on the majority site and −0.01(2) on the minority site, so these were set to unity and zero, respectively, consistent with the MnCaTa_2_O_7_ composition. The NPD data showed no evidence for any other phases in the sample. Plots of the fitted NPD data are shown in Figure 1, with full details of the refined structure of MnCaTa_2_O_7_ are given in the Supporting Information. A representation of the refined structure is shown in Figure 2. It should be noted that, while the tilting distortion of MnCaTa_2_O_7_ is *a*^–^*b*^–^*c*^+^/*b*^–^*a*^–^*c*^+^, it is close to *a*^–^*b*^0^*c*^+^/*b*^0^*a*^–^*c*^+^ as the “*a*” tilt (19.7(1)°) is significantly larger than the “*b*” tilt (1.1(1)°).

### Magnetic Characterization of MnCaTa_2_O_7_

**Figure 3:**
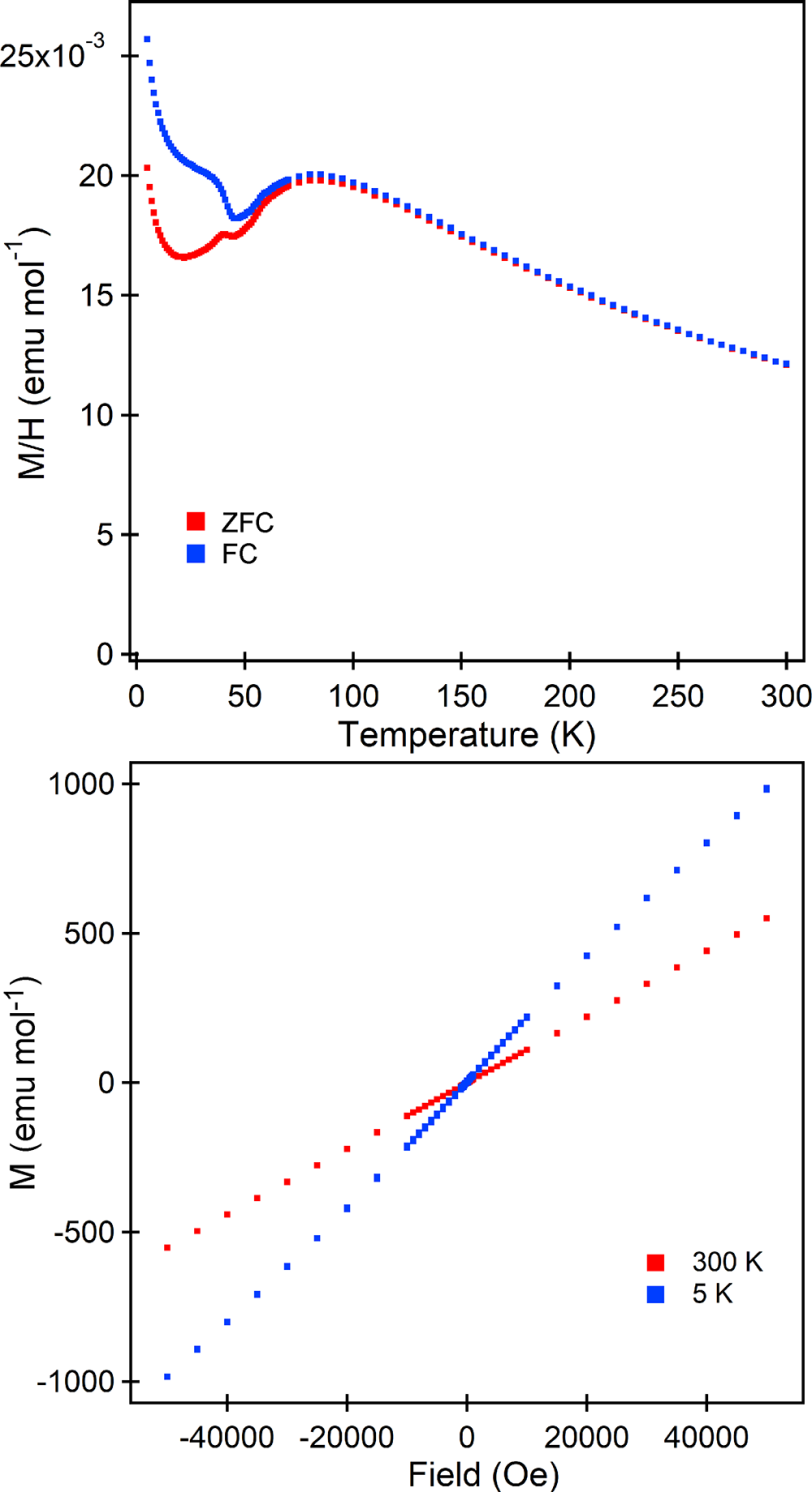
(top) Zero-field-cooled and field-cooled magnetization data collected from MnSrTa_2_O_7_ as a function of temperature in an applied field of 100 Oe. (bottom) Magnetization-field data collected from MnCaTa_2_O_7_ at 300 and 5 K.

Zero-field-cooled (ZFC) and field-cooled (FC) magnetization data were collected from a sample of H_2_-treated MnCaTa_2_O_7_ in an applied field of 100 Oe (Figure 3). These data can be fit by the Curie–Weiss law in the range 165 < *T*/K < 300 to yield values of *C* = 5.829(4) cm^3^ K mol^–1^ and θ = −180.0(3) K, as shown in detail in the Supporting Information. The extracted Curie constant is slightly larger than that expected for an array of *S* = 5/2 Mn^2+^ centers (*C*_expected_ = 4.37 cm^3^ K mol^–1^). On cooling below *T* ∼150 K, the magnetization data exhibit a broad local maximum before the ZFC and FC data diverge below *T* ∼100 K, with the divergence increasing significantly below *T* ∼45 K. Magnetization-field data collected at 300 K are linear and pass through the origin, as shown in Figure 3, while analogous data collected at 5 K, after cooling in an applied field of 50,000 Oe, are slightly sigmoidal but exhibit no significant hysteresis.

**Figure 4:**
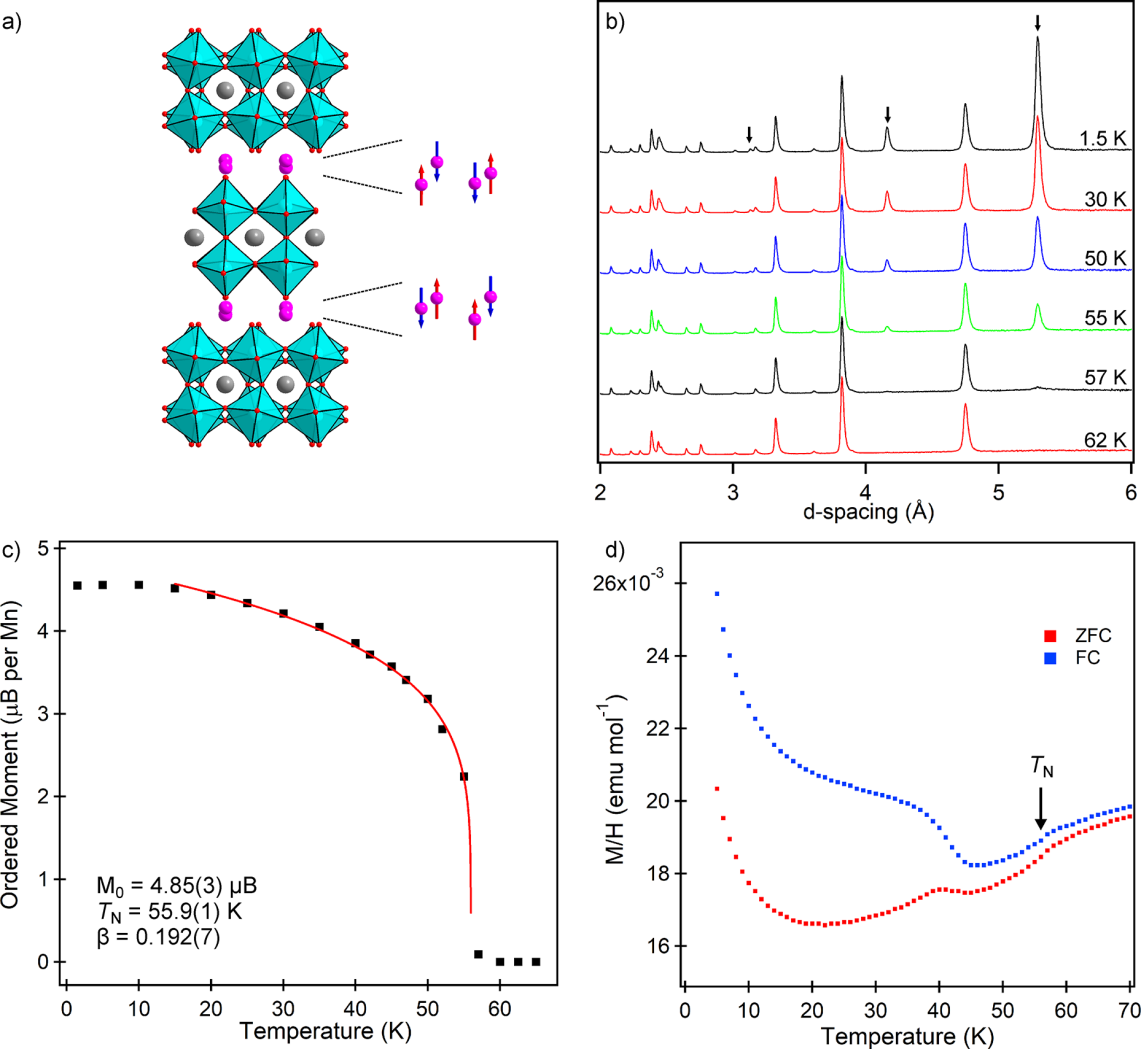
(a) G-type magnetic structure of MnCaTa_2_O_7_. (b) NPD data collected from MnCaTa_2_O_7_ as a function of temperature. Arrows indicate magnetic diffraction peaks. (c) Plot of ordered magnetic moment as a function of temperature, fitted by (*M* = *M*_0_(1 – *T* / *T*_N_)^β^ power law. (d) ZFC and FC magnetization data from MnCaTa_2_O_7_ in range 5 < *T*/K < 70.

NPD data (WISH) collected at 1.5 K from MnCaTa_2_O_7_ exhibit a series of diffraction peaks, not observed in the analogous data collected at room temperature (Figure 4b), which are attributed to a magnetic ordering of the manganese spins. The additional diffraction peaks could be indexed using a single magnetic propagation vector, ***k*** = (0, 0, 0), of the crystallographic unit cell. The structural model (space group *P*2_1_*nm*) was used to generate possible magnetic models, with the help of the ISODISTORT software package,^40,41^ which were refined against the 1.5 K neutron data. The best fit to the data was achieved using a model in magnetic space group *P*2_1_*n*′*m*′, in which Mn spins are aligned parallel to the *z*-axis and adopt a G-type antiferromagnetic ordered arrangement. Refinement of the components of the Mn moments revealed that the magnitude of the ordered moment along *x*- and *y*-directions is zero within error. Therefore, the model was simplified to only allow an ordered moment along the *z*-direction (as shown in Figure 4a), which refined to a value of 4.496(8) μB. Full details of the crystal and magnetic structure are given in the Supporting Information along with the fit to the NPD data.

NPD data (WISH) collected on warming from 1.5 K reveal a decline in the ordered magnetic moment with increasing temperature, which can be fit by a power law (*M* = *M*_0_(1 – *T* / *T*_N_)^β^), as shown in Figure 4c, to yield values of *M*_0_ = 4.85(3) μB, *T*_N_ = 55.9(1) K, β = 0.192(7). Close inspection of the NPD data sets (Figure 4b) reveals that magnetic scattering persists above *T*_N_ (56 K) with the magnetic scattering features broadening significantly with increasing temperature, as shown in detail in the Supporting Information.

## Discussion

Treatment of Li_2_CaTa_2_O_7_ with MnCl_2_ leads to a quantitative, topochemical, Mn-for-Li cation exchange of the host phase resulting in the formation of phase pure MnCaTa_2_O_7_. Diffraction and SHG data reveal that MnCaTa_2_O_7_ adopts a distorted *n* = 2 Ruddlesden-Popper structure, described in the polar space group *P*2_1_*nm*. This distorted structure can be related to an Mn-disordered, undistorted aristotype phase (space group *I*4/*mmm*) by the application of a series of symmetry-lowering distortions with symmetries described by the irreducible representations (irreps) of the parent *I*4/*mmm* space group. Initially, the ordering of the Mn cations into a chequerboard arrangement lowers the symmetry of the phase (and expands the unit cell) consistent with the M_2_^+^ irrep. Subsequently, there are 7 displacive distortions, which are symmetry-allowed on descent from *I*4/*mmm* to *P*2_1_*nm* space group symmetry: Γ_1_^+^, Γ_5_^–^, X_1_^+^, X_2_^+^(0;*a*), X_3_^–^(*b*;*c*), M_2_^+^, and M_5_^–^. However, analysis of the room-temperature structure of MnCaTa_2_O_7_ reveals that only 3 of these allowed displacive distortion modes have significant magnitude: X_3_^–^(*b*;*c*), which principally describes the *a*^–^*b*^–^*c*^0^/*b*^–^*a*^–^*c*^0^ tilting distortion; X_2_^+^(0;*a*), which principally describes the *a*^0^*a*^0^*c*^+^/*a*^0^*a*^0^*c*^+^ tilting distortion; and Γ_5_^–^, which describes a polar cation displacement. The symmetries of these distortion modes are consistent with the trilinear-coupling polar stabilization mechanism, in which the X_3_^–^(*b*;*c*) and X_2_^+^(0;*a*) distortions couple to and stabilize the Γ_5_^–^ polar distortion, as seen in a range of other layered perovskite phases.^15^

It is interesting to note that the parent phase, Li_2_CaTa_2_O_7_, also adopts a polar structure, described in space group *Pna*2_1_, related to the *I*4/*mmm* aristotype structure via the application of an X_3_^–^(*b*;0) *a*^–^*a*^–^*c*^0^/*a*^–^*a*^–^*c*^0^ tilt, an X_2_^+^(0;*a*) *a*^0^*a*^0^*c*^+^/*a*^0^*a*^0^–*c*^+^ tilt and a Γ_3_^–^ polar distortion.^37,38^ However, in this instance, the polar distortion is not stabilized via trilinear coupling to the X_3_^–^(*b*;0) and X_2_^+^(0;*a*) tilts but via an SOJT stabilization mechanism via the TaO_6_ units, as demonstrated by the observation of a phase transition at *T* ∼490 K to a nonpolar phase, described in space group *Pnam*, which retains the X_3_^–^(*b*;0) and X_2_^+^(0;*a*) distortions but not the polar distortion.^37^ Thus, the substitution of Li by Mn retains a noncentrosymmetric framework but changes the mechanism by which polarization is stabilized, suggesting that these two stabilization mechanisms operate on similar energy scales, as observed previously in other layered perovskite systems.^30^

A further comparison can be made between MnCaTa_2_O_7_ and MnSrTa_2_O_7_. Both phases share a common aristotype structure, but in contrast to MnCaTa_2_O_7_, MnSrTa_2_O_7_ adopts a polar structure described in space group *A*2_1_*am* related to the aristotype phase via X_3_^–^(0;*c*) *a*^–^*a*^–^*c*^0^/*a*^–^*a*^–^*c*^0^ and X_2_^+^(0;*a*) *a*^0^*a*^0^*c*^+^/*a*^0^*a*^0^*c*^+^ tilts and a Γ_5_^–^ polar distortion, with the symmetries of these distortions again compatible with a trilinear-coupled stabilization mechanism.^31^ A further difference between the Ca and Sr phases is that the Mn cations in MnSrTa_2_O_7_ exhibit an incommensurately modulated ordering (Y_2_ symmetry), which can be locally described as a chequerboard arrangement, but which inverts filled and vacant positions with a period, which is incommensurate with the underlying SrTa_2_O_7_ framework, in contrast to the long-range commensurate chequerboard order of the Mn cations in MnCaTa_2_O_7_.^31^

The differing octahedral tilts and Mn cation orderings observed for MnCaTa_2_O_7_ and MnSrTa_2_O_7_ can be rationalized by noting that, although chequerboard ordering appears to be the optimum arrangement for minimizing Mn-Mn cation repulsion, the commensurate chequerboard ordering exhibited by MnCaTa_2_O_7_ is not symmetry compatible with the *a*^–^*a*^–^*c*^+^/*a*^–^*a*^–^*c*^+^ distorted, *A*2_1_*am* symmetry framework exhibited by MnSrTa_2_O_7_ as all the pseudo-tetrahedral Mn coordination sites are symmetry equivalent in the *A*2_1_*am* space group. More formally, we can note that the M_2_^+^ irrep, which describes the symmetry lowering due to the chequerboard ordering of Mn cations, is not allowed to have a nonzero magnitude on the descent in symmetry from *I*4/*mmm* to *A*2_1_*am*. Thus, there appears to be a conflict between the optimum octahedral tilting arrangement (*a*^–^*a*^–^*c*^+^/*a*^–^*a*^–^*c*^+^) and the optimum Mn cation ordering (M_2_^+^ chequerboard ordering), with the octahedral tilting “winning” the competition in MnSrTa_2_O_7_ (*a*^–^*a*^–^*c*^+^/*a*^–^*a*^–^*c*^+^ tilt, incommensurate Mn order) and the chequerboard Mn ordering “winning” in MnCaTa_2_O_7_ (*a*^–^*b*^–^*c*^+^/*b*^–^*a*^–^*c*^+^ tilt, chequerboard Mn order), illustrating again that there are a large number of competing ground states in these layered perovskite phases.

The subtle structural differences between MnCaTa_2_O_7_ and MnSrTa_2_O_7_ lead to differences in their physical behavior. As noted previously, on cooling MnSrTa_2_O_7_ adopts G-type antiferromagnetic order (*T*_N_ = 43 K) and on further cooling to *T*_A_ = 38 K, local minima are observed in the *a* and *c* lattice parameters, and discontinuities are observed in the magnitudes of the X_3_^–^(0;*c*) and Γ_5_^–^ distortion modes, accompanied by a large increase in the ferromagnetic component of the magnetic order of the material. The simultaneous change in the magnitudes of both the electrical and magnetic polarizations is taken as a signature of magnetoelectric coupling.^31^

**Figure 5:**
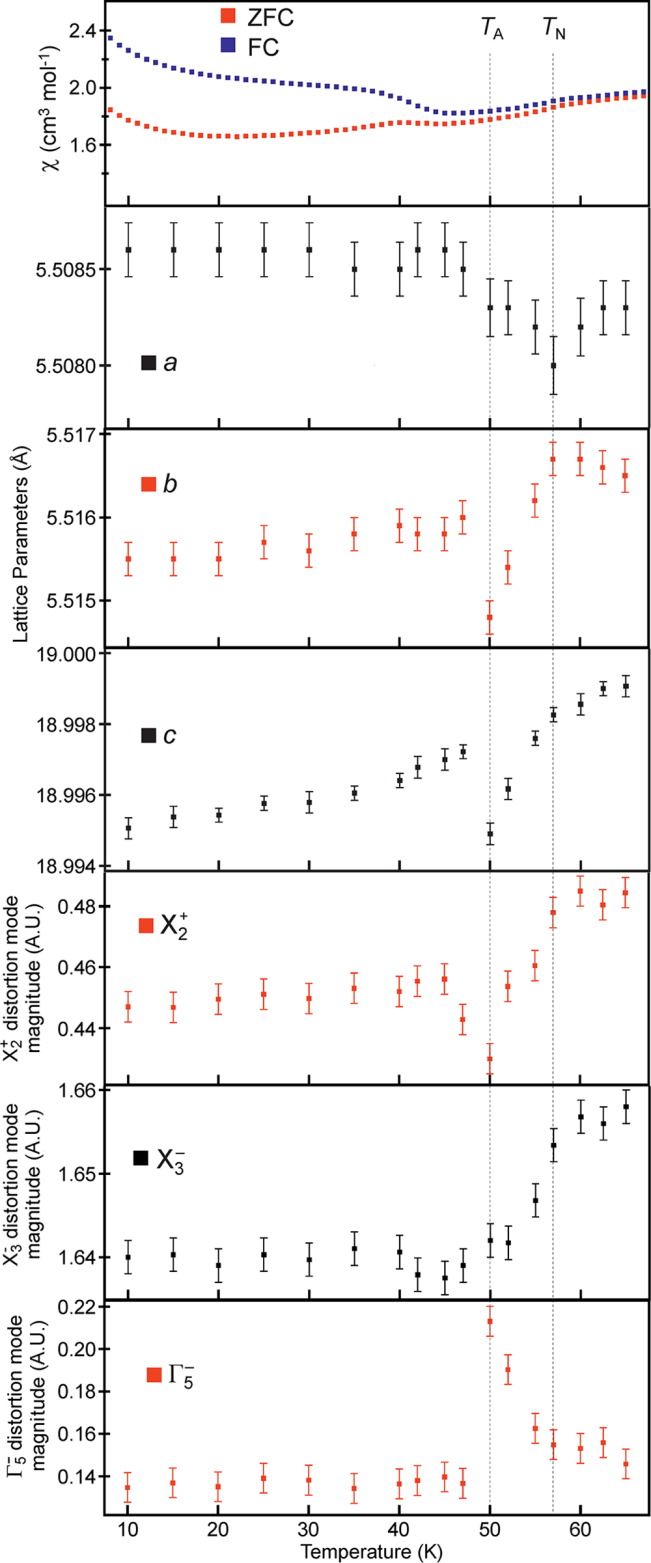
Zero-field cooled and field cooled magnetization data, lattice parameters, X_2_^+^(0;*a*), X_3_^–^(*b*;*c*) and Γ_5_^–^ distortion mode magnitudes (arbitrary units) plotted as a function of temperature.

A similar analysis of MnCaTa_2_O_7_ using the NPD data (WISH) collected as a function of temperature reveals a shallow local minimum in the *a* lattice parameter at *T* = 55 K (i.e., *T*_N_ as determined from the magnetic scattering in the NPD data) and a deeper local minimum in the *b* and *c* lattice parameters at *T*_A_ = 50 K, as shown in Figure 5. Deconvoluting the refined structures of MnCaTa_2_O_7_ determined from these NPD data to extract the magnitudes of the distortion modes reveals that the variation in the X_2_^+^(0;*a*) mode appears to track the variation in the *b* and/or *c* lattice parameters (Figure 5), while the magnitude of the X_3_^–^(*b*;*c*) mode declines monotonically from *T*_N_ to *T*_A_. In contrast, the Γ_5_^–^ mode increases in magnitude by ∼25% on cooling from *T*_N_ to *T*_A_, before sharply returning to its original value below *T*_A_. As shown in Figure 5, there are no dramatic changes in the magnetization data collected from MnCaTa_2_O_7_ at either *T*_N_ or *T*_A_, with no sign of a large increase in the ferromagnetic component of the magnetic moment at *T*_A_ seen for MnSrTa_2_O_7_. Thus, we can conclude that there is no evidence for magnetoelectric coupling in MnCaTa_2_O_7_. The increase in the divergence between the ZFC and FC data seen below *T* ∼40 K is attributed to an impurity phase in the sample, likely Mn_3_O_4_, which cannot be observed by diffraction.

The differing structural and magnetic behavior of MnCaT_2_O_7_ and MnSrTa_2_O_7_ around their respective magnetic ordering temperatures is surprisingly given the apparent similarity of the phases. Both derive from identical Mn*A*Ta_2_O_7_ aristotype parent structures, both adopt polar structural distortions with the polarization aligned parallel to the *x*-axis, both adopt magnetic order with structures described in magnetic space groups, which allow net ferromagnetic moments aligned parallel to the *x*-axis, yet the magnetic and electric polarizations appear to couple in MnSrTa_2_O_7_ and not in MnCaTa_2_O_7_. The implication is that the differences in physical behavior arise from the subtle differences between the two phases.

The most obvious difference between the two phases is the combination of Mn cation-order and octahedral tilting distortion each exhibits: M_2_^+^ chequerboard Mn-order and an X_3_^–^(*b*;*c*) *a*^–^*b*^–^*c*^0^/*b*^–^*a*^–^*c*^0^ tilt for MnCaTa_2_O_7_ compared to Y_2_ incommensurate Mn-order and an X_3_^–^(0;*c*) *a*^–^*a*^–^*c*^0^/*a*^–^*a*^–^*c*^0^ tilting distortion for MnSrTa_2_O_7_. However, these features of the materials do not appear to be immediately relevant to the appearance of a ferromagnetic polarization as our previous symmetry analysis of the magnetoelectric coupling in MnSrTa_2_O_7_ revealed that it is coupling between the X_2_^+^(0;*a*) *a*^0^*a*^0^*c*^+^/*a*^0^*a*^0^*c*^+^ tilt and the *m*M_3_^+^ G-type magnetic order, which generates and stabilizes the *m*Γ_5_^+^ ferromagnetic polarization of the phase.^31^

However, given that both MnSrTa_2_O_7_ and MnCaTa_2_O_7_ exhibit X_2_^+^(0;*a*) tilts and *m*M_3_^+^ magnetic order in their ground states, but only MnSrTa_2_O_7_ exhibits a significant ferromagnetic polarization, we can conclude that, while symmetry allowed, the coupling between X_2_^+^(0;*a*) and *m*M_3_^+^ modes in MnCaTa_2_O_7_ is much weaker than in MnSrTa_2_O_7_. The origin of the weak X_2_^+^(0;*a*)/*m*M_3_^+^ coupling in MnCaTa_2_O_7_ is not clear. It does not appear to be due to the small magnitude of the X_2_^+^(0;*a*) tilt in the calcium phase (∼ 2.5 °), as the corresponding tilt in the strontium phase is also small (∼1.5 °). Clearly, this issue warrants further attention and further highlights the complexities of the distortion mode couplings in this class of material.

## Conclusions

Reaction between Li_2_CaTa_2_O_7_ and MnCl_2_ allows the preparation of the metastable phase MnCaTa_2_O_7_. Detailed structural characterization reveals MnCaTa_2_O_7_ adopts a layered perovskite structure in which the CaTa_2_O_7_ layers adopt an *a*^–^*b*^–^*c*^+^/*b*^–^*a*^–^*c*^+^ tilting distortion with the Mn^2+^ cations ordered in a chequerboard manner in the interlayer region. SHG data confirm that a noncentrosymmetric structure is adopted by MnCaTa_2_O_7_, which NPD data reveal is described in the space group *P*2_1_*nm*. Symmetry analysis shows the experimental structure can be related to an undistorted aristotype structure (space group *I*4/*mmm*) via two tilting distortion modes X_3_^–^(*b*;*c*), which principally describes the *a*^–^*b*^–^*c*^0^/*b*^–^*a*^–^*c*^0^ tilting distortion and X_2_^+^(0;*a*), which principally describes the *a*^0^*a*^0^*c*^+^/*a*^0^*a*^0^*c*^+^ tilting distortion. These two tilting distortions appear to stabilize a polar Γ_5_^–^ distortion via the trilinear-coupled hybrid improper stabilization mechanism. On cooling, MnCaTa_2_O_7_ adopts a G-type antiferromagnetically ordered structure below *T*_N_ = 56 K and exhibits large lattice parameter anomalies, especially in the *b* and *c* lattice parameters, at *T*_A_ = 50 K. However, in contrast to MnSrTa_2_O_7_, there is no change to the magnetization at *T*_A_ and no evidence for weak ferromagnetism in MnCaTa_2_O_7_ at any temperature.

## Supporting Information Available

Detailed description of the structural characterization of Li_2_CaTa_2_O_7_ and MnCaTa_2_O_7_ at room temperature, and structural and magnetic characterization of MnCaTa_2_O_7_ at low temperature (PDF)
