# Supplementary material for: MnCaTa2O7—A Magnetically Ordered Polar Phase Prepared via Cation Exchange
Source: Chem Mater. 2023 Sep 9;35(18):7839–46. doi: 10.1021/acs.chemmater.3c01850 (PMC10538500; doi:10.1021/acs.chemmater.3c01850)
Supplement: Supplementary file 1 — cm3c01850_si_001.pdf [file cm3c01850_si_001.pdf]

$MnCaTa_2O_7$ – a magnetically ordered polar phase prepared via cation exchange.

Subhadip Mallick, Fabio Orlandi, Pascal Manuel, Weiguo Zhang,
P. Shiv Halasyamani, and Michael A. Hayward*

Supporting Information

Table of Contents

1. Structural Characterisation of $Li_2CaTa_2O_7$

Figure S1. Observed, calculated and difference plots from the structural refinement of $Li_2CaTa_2O_7$ against SXRD data collected at room temperature.

Table S1. Parameters from the structural refinement of $Li_2CaTa_2O_7$ against the SXRD data collected at room temperature.

2. Structural Characterisation of $MnCaTa_2O_7$

Figure S2. Observed calculated and difference plots from the structural refinement of $MnCaTa_2O_7$ against NPD data collected at room temperature.

Table S2. Parameters from the structural refinement of $MnCaTa_2O_7$ against NPD data collected at room temperature.

3. Magnetic Characterisation of $MnCaTa_2O_7$

Figure S3. Plot of inverse susceptibility against temperature for $MnCaTa_2O_7$, measured in an applied field of 100 Oe.

Figure S4. Observed calculated and difference plots from the structural and magnetic refinement of $MnCaTa_2O_7$ against NPD data collected at 1.5 K.

Figure S4. Observed calculated and difference plots from the structural and magnetic refinement of $MnCaTa_2O_7$ against NPD data collected at 1.5 K using the 5 detector banks of the WISH diffractometer.

Figure S5. NPD data collected from $MnCaTa_2O_7$ showing diffuse magnetic scattering above T_N (56 K).

Table S3. Crystal and magnetic structural parameters from the refinement of $MnCaTa_2O_7$ against NPD data (WISH) collected at 1.5 K.

1. Structural Characterisation of $Li_2CaTa_2O_7$

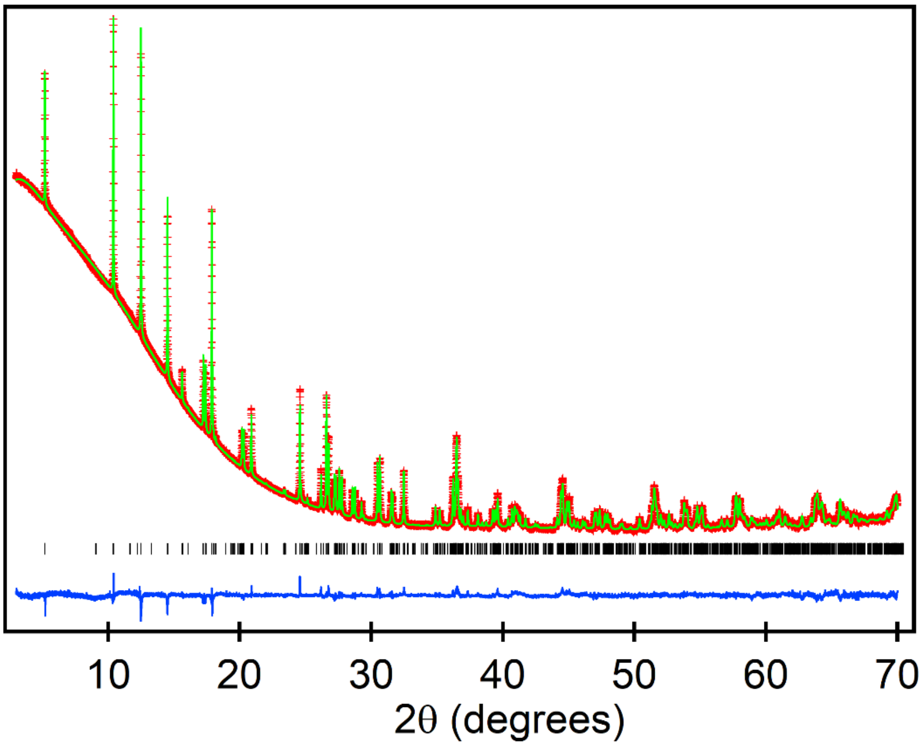

Figure S1. Observed, calculated and difference plots from the structural refinement of $Li_2CaTa_2O_7$ against SXRD data collected at room temperature.

Atom	Site	*x*	*y*	*z*	Occ.	B_{eq} (Å^2)
Li1*	4*a*	0.003	0.244	0.515	1	0.39(13)
Li2*	4*a*	0.985	0.745	0.491	1	0.39(13)
Ca	4*a*	0.281(1)	0.515(1)	0.0.244(1)	1	0.71(9)
Ta1	4*a*	0.7368(4)	0.9968(5)	0.863(1)	1	0.17(8)
Ta2	4*a*	0.2572(7)	0.0154(3)	0.139(1)	1	0.23(9)
O1*	4*a*	0.731	0.5764	0.2508	1	0.30(6)
O2*	4*a*	0.247	0.511	0.5380	1	1.72(17)
O3*	4*a*	0.757	0.454	0.4646	1	0.26(13)
O4*	4*a*	0.461	0.714	0.1352	1	0.51(14)
O5*	4*a*	0.543	0.294	0.8703	1	0.43(8)
O6*	4*a*	0.969	0.714	0.8343	1	0.40(14)
O7*	4*a*	0.022	0.273	0.1635	1	0.59(13)
$Li_2CaTa_2O_7$ – space group *Pna*2_1 (#33)<br>*a* = 5.51225(3) Å, *b* = 5.46373(3) Å, *c* = 18.2321(1) Å, volume = 549.105(6) Å^3<br>Formula weight = 527.85 g mol^{-1}, Z = 4<br>Radiation source: Synchrotron X-ray, λ = 0.8268 Å<br>Temperature: 298 K<br>$_{w}R_{p}$ = 1.32 % R_{Bragg} = 1.43 %						

Table S1. Parameters from the structural refinement of $Li_2CaTa_2O_7$ against the SXRD data collected at room temperature. Atomic positions of atoms marked with * were not refined due to poor X-ray scattering power and were fixed at values reported in *Inorg. Chem.* 2016, **55**, 2309-2323.

2. Structural Characterisation of $MnCaTa_2O_7$

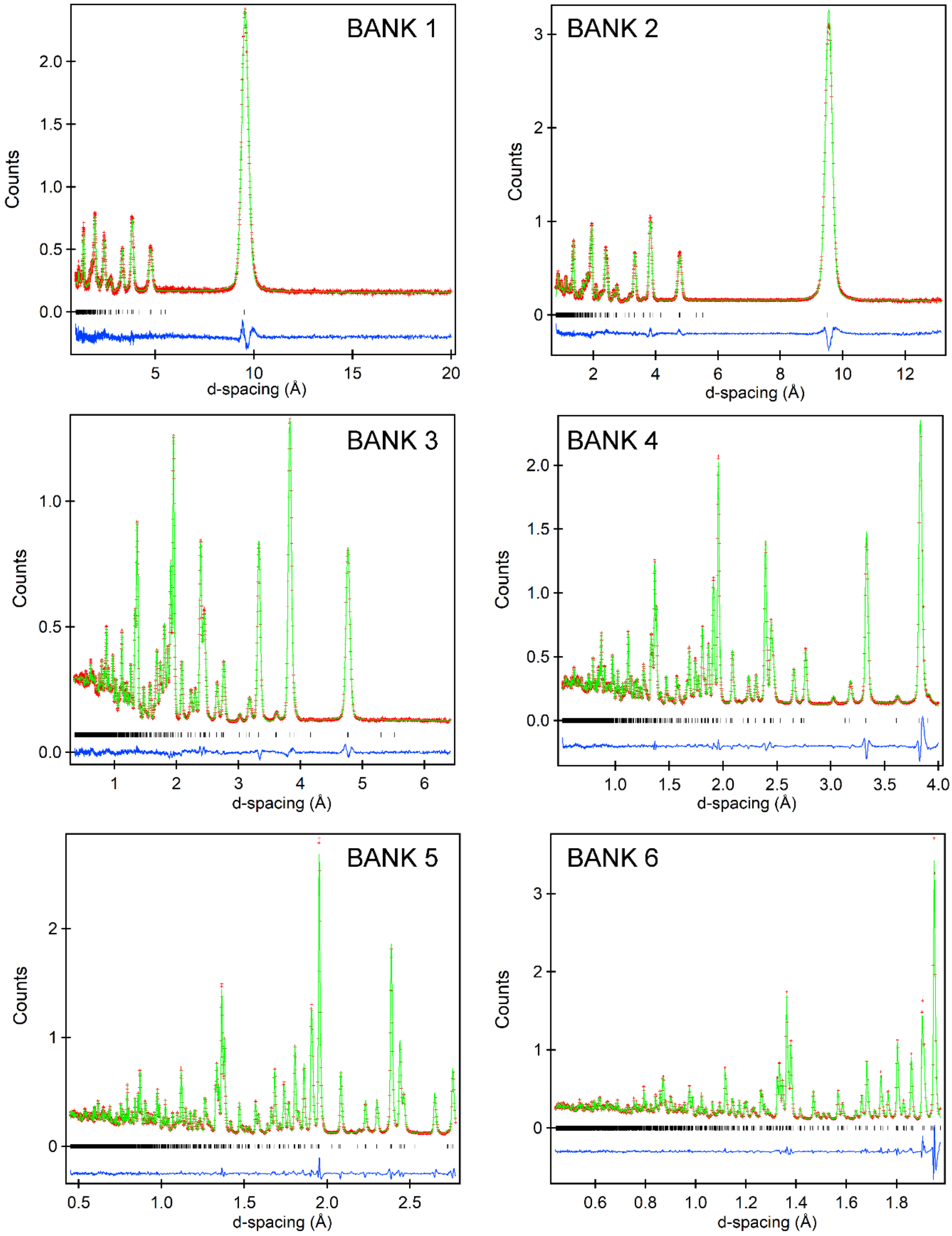

Figure S2. Observed calculated and difference plots from the structural refinement of $MnCaTa_2O_7$ against NPD data collected at room temperature using the 6 detector banks of the GEM diffractometer.

Atom	Site	*x*	*y*	*z*	Occ.	B_{iso} (Å^2)
Mn1	4*b*	0.2375(15)	0.2515(10)	0.7382(1)	1	0.85(3)
Ca1	2*a*	0.9893(11)	0.9990(10)	0	1	0.23(4)
Ca2	2*a*	0.5108(18)	0.5152(15)	0	1	1.69(9)
Ta1	4*b*	0.9966(7)	0.9964(5)	0.3940(1)	1	0.23(4)
Ta2	4*b*	0.5025(6)	0.4997(5)	0.3939(1)	1	0.27(4)
O1	2*a*	0.5463(7)	0.9313(7)	0	1	0.48(6)
O2	2*a*	0.9351(10)	0.5497(8)	0	1	0.68(7)
O3	4*b*	0.7301(8)	0.2654(6)	0.0886(1)	1	0.37(4)
O4	4*b*	0.2454(13)	0.7491(7)	0.0932(1)	1	1.10(5)
O5	4*b*	0.2599(7)	0.2359(7)	0.6213(1)	1	0.88(2)
O6	4*b*	0.7381(10)	0.7569(8)	0.5797(1)	1	0.62(2)
O7	4*b*	0.9647(6)	0.9720(7)	0.2957(3)	1	0.68(5)
O8	4*b*	0.5183(7)	0.5349(7)	0.2967(3)	1	0.92(6)
$MnCaTa_2O_7$ – space group $P2_1nm$ (#31)<br>a = 5.5155(4) Å, b = 5.5169(4) Å, c = 19.0303(12) Å,<br>volume = 579.07(6) Å^3<br>Formula weight = 568.91 g mol^{-1}, Z = 4<br>Radiation source: Neutron Time of Flight<br>Temperature: 298 K<br>R_p = 3.088 %, $_wR_p$ = 2.879 %						

Table S2. Parameters from the structural refinement of $MnCaTa_2O_7$ against NPD data collected at room temperature.

3. Magnetic Characterisation of $MnCaTa_2O_7$

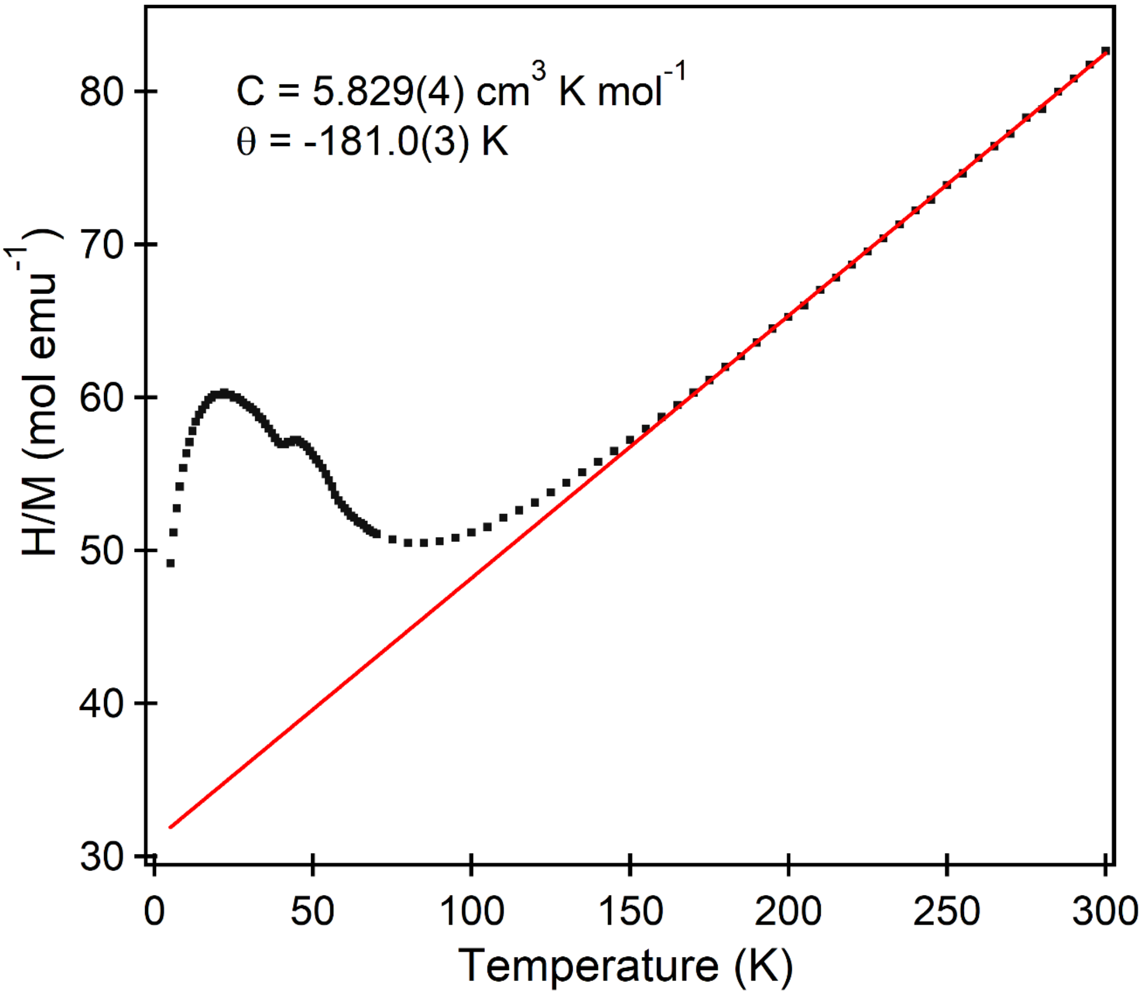

Figure S3. Plot of inverse susceptibility against temperature for $MnCaTa_2O_7$, measured in an applied field of 100 Oe. Linear fit in the range 165 < *T*/K < 300 is consistent with Curie-Weiss behaviour yielding C = 5.829(4) cm^3 K mol^{-1} and θ = 181.0(3) K.

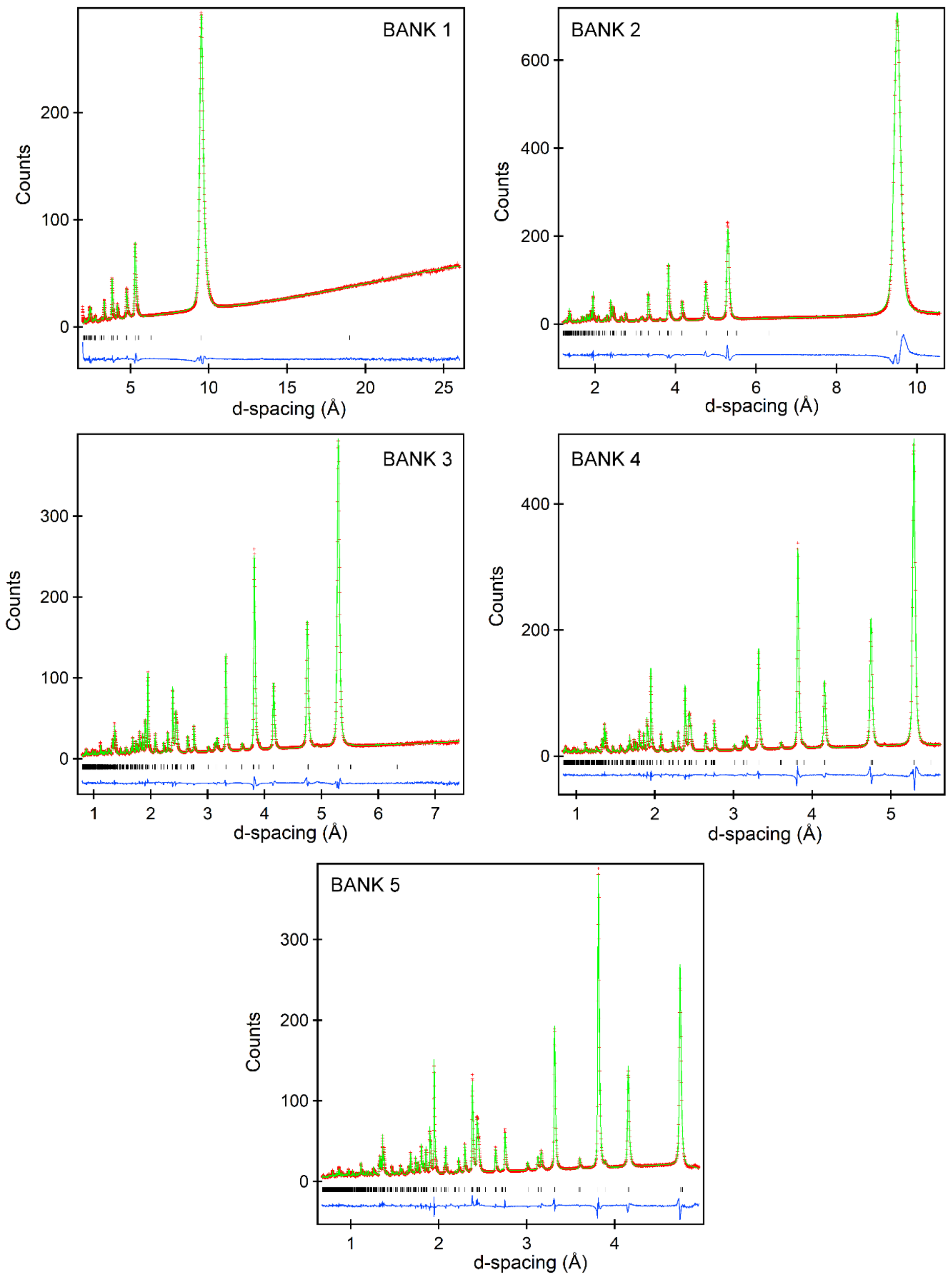

Figure S4. Observed calculated and difference plots from the structural and magnetic refinement of $MnCaTa_2O_7$ against NPD data collected at 1.5 K using the 5 detector banks of the WISH diffractometer.

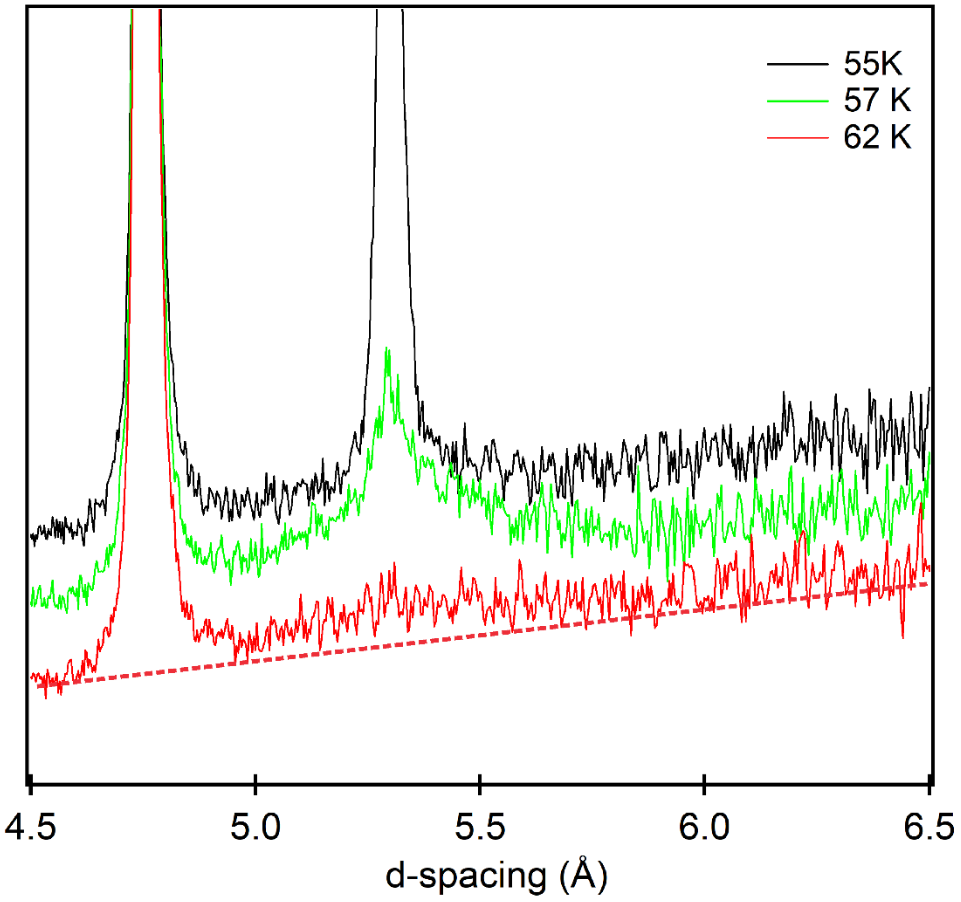

Figure S5. NPD data collected from $MnCaTa_2O_7$ showing diffuse magnetic scattering above T_N (56 K).

Atom	Site	*x*	*y*	*z*	Occ.	B_{iso} (Å^2)
Mn1	4*b*	0.2411(12)	0.2530(8)	0.7377(1)	1	0.34(2)
		M_x = 0	M_y = 0	M_z = 4.496(8)		
Ca1	2*a*	0.9919(17)	0.9906(17)	0	1	0.12(4)
Ca2	2*a*	0.5060(20)	0.5110(20)	0	1	0.85(7)
Ta1	4*b*	0.9928(8)	0.9948(8)	0.3939(1)	1	0.12(3)
Ta2	4*b*	0.4996(9)	0.5015(8)	0.3938(1)	1	0.06(3)
O1	2*a*	0.5511(10)	0.9346(11)	0	1	0.33(5)
O2	2*a*	0.9442(11)	0.5627(10)	0	1	0.46(6)
O3	4*b*	0.7302(8)	0.2661(8)	0.0894(2)	1	0.32(5)
O4	4*b*	0.2571(8)	0.7378(9)	0.0914(2)	1	0.73(6)
O5	4*b*	0.2653(7)	0.2321(6)	0.6215(5)	1	0.57(2)
O6	4*b*	0.7345(8)	0.7613(8)	0.5796(1)	1	0.40(2)
O7	4*b*	0.9624(10)	0.9760(11)	0.2959(2)	1	0.45(5)
O8	4*b*	0.5207(10)	0.5336(10)	0.2955(2)	1	0.47(5)
$MnCaTa_2O_7$ – space group $P2_1n'm'$ (#31.127)<br>a = 5.5086(4) Å, b = 5.5107(4) Å, c = 18.9905(11) Å,<br>volume = 576.48(6) Å^3<br>Formula weight = 568.91 g mol^{-1}, Z = 4<br>Radiation source: Neutron Time of Flight<br>Temperature: 1.5 K<br>R_p = 1.991 %, $_wR_p$ = 2.333 %						

Table S3. Crystal and magnetic structural parameters from the refinement of $MnCaTa_2O_7$ against NPD data (WISH) collected at 1.5 K.
